# Establishment of an Indirect ELISA Detection Method for Porcine Circovirus 3 Based on Soluble Cap Protein

**DOI:** 10.3390/vetsci13070704

**Published:** 2026-07-18

**Authors:** Weizhen Shen, Mengran Zhang, Yixiang Lian, Jiahui Xu, Yunye Jiang, Jing Chen, Jin Cui, Bin Zhou

**Affiliations:** 1 MOE Joint International Research Laboratory of Animal Health and Food Safety, College of Veterinary Medicine, Nanjing Agricultural University, Nanjing 210095, China; 15057302027@163.com (W.S.); meng_ran_zhang@163.com (M.Z.); f811206291@hotmail.com (Y.L.); 9231710726@njau.edu.cn (J.X.); 1381392029@163.com (Y.J.); t2025065@njau.edu.cn (J.C.); 2 Key Laboratory of Animal Bacteriology, Ministry of Agriculture and Rural Affairs, Nanjing Agricultural University, Nanjing 210095, China; 3 College of Veterinary Medicine, Northeast Agricultural University, 600 Changjiang Street Xiangfang District, Harbin 150030, China; jincui@neau.edu.cn; 4 Key Laboratory of Diagnosis and Treatment for Epizootic Diseases of Animals in Cold Regions of Heilongjiang Province, Northeast Agricultural University, 600 Changjiang Street Xiangfang District, Harbin 150030, China

**Keywords:** porcine circovirus 3, capsid protein, prokaryotic expression system, soluble expression, indirect ELISA, swine reproductive disorders

## Abstract

Porcine circovirus 3 (PCV3) is an emerging swine pathogen associated with reproductive disorders, multisystemic inflammation, and other clinical syndromes. The capsid protein (Cap), the principal structural and immunogenic component, is an ideal antigen for serological detection. However, its poor solubility in prokaryotic expression systems limits diagnostic assay development. In this study, the N-terminal 32 amino acids of Cap were truncated, and the protein was expressed in a soluble form using the SHuffle T7 Express strain. An indirect enzyme-linked immunosorbent assay (ELISA) using purified soluble Cap as a coating antigen was established and optimized. The assay exhibited excellent specificity, with no cross-reactivity against other common swine pathogens, high sensitivity, and good repeatability, with intra- and inter-assay coefficients of variation below 10%. The cut-off value was determined, and testing of 144 porcine serum samples showed a 78.47% concordance rate with PCR, indicating good diagnostic performance. This Cap-based indirect ELISA provides a reliable, cost-effective, and high-throughput tool for large-scale serological surveillance and epidemiological monitoring of PCV3, supporting improved disease control strategies in pig populations.

## Simple Summary

In this study, an indirect enzyme-linked immunosorbent assay (ELISA) was established for the detection of antibodies against porcine circovirus 3 (PCV3), based on a soluble capsid protein (Cap) expressed in *Escherichia coli*. In conventional prokaryotic expression systems, the Cap is predominantly produced as insoluble inclusion bodies, thereby increasing antigen production costs and complicating serological detection procedures. In the present work, soluble expression of the Cap was successfully achieved through sequence-guided truncation, the optimization of induction conditions, and the selection of appropriate expression strains. Furthermore, a sensitive and stable indirect ELISA method was developed, providing a reliable tool for large-scale serological investigation of PCV3.

## 1. Introduction

Porcine circovirus 3 (PCV3), a member of the genus *Circovirus* within the family *Circoviridae*, is a non-enveloped, single-stranded DNA virus that has emerged as a globally prevalent swine pathogen [1]. First identified in pigs affected by porcine dermatitis and nephropathy syndrome in the United States in 2016 [2], PCV3 has subsequently been detected across Asia, Europe, North America, and South America, highlighting its extensive geographical spread and growing epidemiological significance [3]. A growing body of evidence has linked PCV3 infection to a diverse range of clinical conditions, including reproductive failure, porcine dermatitis and nephropathy syndrome, multisystemic inflammation, respiratory disorders, and impaired growth performance, collectively resulting in considerable economic losses to the global swine industry [4]. Consequently, the development of rapid, reliable, and high-throughput diagnostic platforms is of paramount importance for disease surveillance, risk assessment, and the implementation of effective control strategies [5,6,7].

Current diagnostic methodologies for PCV3 can be broadly categorized into molecular and serological approaches. Nucleic acid-based techniques, including conventional PCR and quantitative real-time PCR (qPCR) [8], are widely utilized for the detection of active viral infection. Nevertheless, these methods provide limited information regarding herd immunity, previous exposure, or population-level infection dynamics [7]. In contrast, serological assays facilitate the evaluation of cumulative viral exposure and antibody prevalence, making them particularly valuable for large-scale epidemiological investigations and long-term surveillance programs. The capsid protein (Cap), the sole structural protein and major immunogenic determinant of PCV3, contains multiple immunoreactive B-cell epitopes capable of eliciting robust virus-specific antibody responses [7]. Owing to these characteristics, the Cap is widely recognized as the antigen of choice for the development of serological diagnostic assays [9].

Numerous studies have reported that the PCV3 Cap expressed in conventional prokaryotic systems, particularly *Escherichia coli* (*E. coli*) BL21 (DE3), is predominantly produced as insoluble inclusion bodies, thereby limiting its utility in serological assay development [10,11]. Bioinformatic analyses have revealed that the N-terminal region of the Cap contains a nuclear localization sequence (NLS) enriched in arginine residues and rare codons, features that are believed to impede proper protein folding and contribute to its poor solubility [12]. Although eukaryotic expression systems can generate soluble Cap, their application is often constrained by high production costs, low expression yields, and labor-intensive procedures, which collectively hinder large-scale implementation [13,14]. Therefore, the development of a cost-effective and scalable serological assay based on soluble recombinant Cap remains highly desirable.

In the present study, the sequence characteristics of Cap were systematically analyzed using bioinformatic approaches, and a truncated variant was designed. The truncated gene was subsequently cloned into the pET-28a expression vector and expressed in engineered strains (*E. coli*) to facilitate the efficient production of soluble recombinant protein. Using the purified soluble Cap as the coating antigen, an indirect enzyme-linked immunosorbent assay (ELISA) was established and optimized for the detection of PCV3-specific antibodies. Furthermore, the assay was comprehensively evaluated in terms of specificity, sensitivity, reproducibility, and clinical applicability. The overarching goal was to provide a reliable, cost-effective, high-throughput, and practical indirect ELISA for large-scale serological surveillance and epidemiological investigations of PCV3 infection.

## 2. Materials and Methods

### 2.1. Bacterial and Serum Samples

PCV3 nucleic acid (GenBank No. PP927827.1) was preserved in the laboratory; pET-28a (+) plasmid, *E. coli* DH5α and *E. coli* BL21 (DE3) (Vazyme Biotech Co., Ltd., Nanjing, China), SHuffle T7 Express *E. coli* B, *E. coli* Lemo21 (DE3) and *E. coli* Rosetta- gami2 (DE3) pLysS (Weidi Biotechnology Co., Ltd., Shanghai, China); Positive sera for porcine circovirus 2 (PCV2), classical swine fever virus (CSFV), porcine reproductive and respiratory syndrome virus (PRRSV), Japanese encephalitis virus (JEV), African swine fever virus (ASFV), pseudorabies virus (PRV) and foot-and-mouth disease Virus (FMDV) all come from commercial ELISA test kits; Forty PCV3-positive serum and thirty PCV3-negative serum samples were stored and validated using qPCR [15,16]; A total of 144 pig serum samples were collected from multiple commercial pig farms located in Jiangsu Province, China. These samples were obtained from pigs of various ages (including piglets, growing pigs, and sows) raised under intensive production systems. All samples were stored at −80 °C before use.

### 2.2. Sequence Feature Analysis and Structure Prediction

Downloaded 2403 PCV3 Cap sequences uploaded from 2016 to 2024 from the NCBI database (http://www.ncbi.nlm.nih.gov) and analyzed the conservation of these sequences using MEGA11.0 software [17] and the Weblogo 3 website [18]. The conserved PCV3 Cap sequences obtained were used to predict the nuclear localization relevance of amino acid residues via the NLS Explorer website [19], B-cell epitopes via the BepiPred 3.0 website [20], and transmembrane regions via the TMHMM 2.0 website [21]. After the above analysis, the first 32 aa were removed, and the structures of truncated Cap monomers and multimers were predicted via the AlphaFold 3 website [22].

### 2.3. Cap Gene Amplification and Cloning

Using the Cap gene sequence of the PCV3 nucleic acid (GenBank No. PP927827.1) whose sequencing results are consistent with the conserved sequence as a reference, primers were designed to incorporate homologous sequences at both ends of the pET-28a (+) vector: Cap-F: 5′-atgggtcgcggatccATGCCCACAG CTGGCACATACT-3′; Cap-R: 5′-gtggtggtggtggtgTTAGAGAACGGACTTGTAACGA-3′ (Homologous sequence lowercase letters), and primer protective sites, restriction sites and anti-migratory sites were also considered in the design. The truncated *Cap* was amplified using 2 × Phanta Flash Master Mix (Vazyme Biotech Co., Ltd., Nanjing, China) in a 50 μL reaction containing 500 ng DNA, 2 μL each primer. Cycling: 98 °C for 30 s; 35 cycles of 98 °C for 10 s, 63 °C for 5 s, 72 °C for 20 s; final 72 °C for 1 min. The product was cloned into *EcoR I*/*Xho I* (Takara Bio Inc., Dalian, China)-digested pET-28a via Seamless Cloning (Vazyme Biotech Co., Ltd., Nanjing, China). After transformation into DH5α, positive clones (Verified by PCR with T7/T7ter primers) were selected on Luria–Bertani (LB) agar plates supplemented with 50 mg/L kanamycin and verified by PCR with T7/T7ter primers and restriction digestion. Following validation by Sangon Biotech Co., Ltd. (Shanghai, China), the correctly sequenced recombinant plasmids were designated pET-28a-PCV3-Cap.

### 2.4. Soluble Expression, Purification, and Validation of the Cap

Both the recombinant plasmid pET-28a-PCV3-Cap and the empty vector were separately introduced into BL21 (DE3). Positive transformants were grown in LB agar plates supplemented with 50 μg/mL kanamycin overnight at 37 °C, then expanded at a 1:100 dilution until they reached an OD_600_ nm of approximately 0.6. Cap expression was induced with Isopropyl *β*-D-1-thiogalactoside (IPTG) (Beyotime Biotech Inc., Shanghai, China) at 16 °C. IPTG concentrations (0.2, 0.4, 0.8 mmol/L) and durations (16, 20, 24 h) were tested to find optimal conditions. Bacterial pellets were harvested and lysed by sonication, and the soluble and insoluble fractions were analyzed by SDS-PAGE (Solarbio Co., Ltd., Beijing, China) to determine Cap solubility in BL21 (DE3). The plasmid pET-28a-PCV3-Cap was then introduced into SHuffle T7 Express, Lemo21 (DE3), and Rosetta-gami2 (DE3) pLysS strains, induced under the optimized conditions, and the predominant expression modality of Cap was assessed by SDS-PAGE and Western blotting.

The recombinant protein in the supernatant was purified using a His Trap™ FF column (Smart-Lifesciences Biotechnology Co., Ltd., Changzhou, China), and the target protein was then eluted with elution buffer containing 200 mmol/L imidazole. The eluate was concentrated and desalted by centrifugal filtration, and protein concentration was determined by BCA assay (Beyotime Biotech Inc., Shanghai, China). The reactivity of recombinant Cap was identified by Western blotting.

### 2.5. Western Blotting

Protein samples were separated on 12.5% SDS-PAGE and transferred to PVDF membranes (Beyotime Biotech Inc., Shanghai, China) at 300 mA for 1 h. Membranes were blocked with 5% skim milk in PBST (8.0 g NaCl, 0.2 g KCl, 2.9 g Na_2_HPO_4_·12H_2_O, 0.2 g KH_2_PO_4_, 0.5 mL Tween-20 per L) for 1 h at room temperature. Primary antibodies (PCV3-positive serum 1:1000, His-tag mAb 1:1000, SPF serum 1:1000, PCV2-positive serum 1:1000, all in 5% BSA, diluted in PBS) were incubated overnight at 4 °C. HRP-conjugated goat anti-pig or anti-mouse IgG (1:5000 in 5% skim milk, diluted in PBST) was applied for 1 h at room temperature. After PBST washes, signals were detected with ECL (Beyotime Biotech Inc., Shanghai, China) and imaged.

### 2.6. Optimization of Antigen Coating Concentration and Serum Dilution

Checkerboard titrations were performed to determine optimal coating antigen concentration and serum dilution. The purified Cap was serially diluted (0.25–8.00 mg/L) in carbonate buffer solution (CBS) and coated (100 μL/well) overnight at 4 °C. Plates were washed with PBST, blocked with 5% skim milk (200 μL/well) for 2 h at 37 °C. PCV3-positive and -negative sera were diluted from 1:25 to 1:3200 and incubated (100 μL/well) for 2 h at 37 °C. After washing, HRP-conjugated goat anti-pig IgG (1:5000) was added for 1 h at 37 °C. TMB substrate (50 μL/well) was added for 15 min at 37 °C, stopped with 50 μL stop solution, and OD_450_ was measured.

### 2.7. Optimization of ELISA Experimental Conditions

After determining the antigen coating concentration and serum dilution, coating buffers (CBS, PBS, methanol [23]), blocking conditions (2.5% BSA, 5% skim milk, 10% FBS; 2 h at 37 °C or overnight at 4 °C), primary antibody incubation time (0.5, 1, 2 h), secondary antibody dilution (1:2500–1:20,000), secondary antibody incubation time (15, 30, 60 min), and color development time (5, 10, 20 min) were sequentially optimized.

### 2.8. Determination of Cut-Off Value for Indirect ELISA

To establish the cut-off value for the indirect ELISA method, seventy well- characterized sera (40 PCV3-positive, 30 negative, confirmed by qPCR) were tested with the optimized ELISA. Receiver operating characteristic (ROC) curve analysis was performed using GraphPad Prism 11.0 (GraphPad Inc., San Diego, USA) [24]. The Youden index was derived from sensitivity and specificity, and the OD_450_ value corresponding to the maximum Youden index was selected as the diagnostic cut-off value.

### 2.9. Evaluation of Assay Specificity, Sensitivity, and Reproducibility

Under optimized conditions, sera positive for CSFV, PRRSV, JEV, PCV2, FMDV, PRV, and ASFV were tested to evaluate potential cross-reactivity and specificity. Sensitivity was assessed by determining the maximum dilution of PCV3-positive serum using 2-fold serial dilutions from 1:200 to 1:25,600. Precision was evaluated by intra- and inter-batch tests: three positive and three negative sera were assayed in triplicate on the same batch of coated plates (intra-batch), and the same six sera were tested on plates from different batches (inter-batch), with coefficients of variation (CVs) calculated for both.

### 2.10. Evaluation with Clinical Samples

A total of 144 pig serum samples was analyzed using both the established indirect ELISA and the Chinese regional PCV3 standard PCR method (DB37/T 4047—2020) [25]. The results were analyzed, and the agreement rate was calculated. Since PCR detects viral nucleic acid while ELISA detects host antibodies, the two methods target biologically distinct markers, and their results are not directly equivalent. The agreement rate between the two methods was calculated to evaluate the clinical utility of the developed ELISA.

### 2.11. Statistical Analysis

All experimental data were analyzed using two-tailed Student’s *t*-tests in GraphPad Prism 11.0 software [24]. Receiver operating characteristic (ROC) curve analysis was employed to determine the optimal diagnostic cutoff value for the indirect ELISA. The Youden index (sensitivity + specificity − 1) was calculated to select the cutoff value that maximized diagnostic performance. The area under the ROC curve (AUC) was reported with its 95% confidence interval and corresponding *p* value to evaluate overall assay accuracy. Sensitivity and specificity were calculated with 95% confidence intervals. For reproducibility assessments, the OD_450_ from intra-assay and inter-assay experiments was used to calculate the mean ($\bar{X}$), standard deviation (SD), and coefficient of variation (CV, expressed as a percentage). A CV below 10% was considered indicative of acceptable reproducibility. The agreement was calculated as follows: Agreement = (number of true-positive/true-negative samples)/(total number of positive/negative samples). All experiments were performed in triplicate unless otherwise specified.

## 3. Results

### 3.1. Sequence Analysis and Structure Prediction of Cap

**Figure 1:**
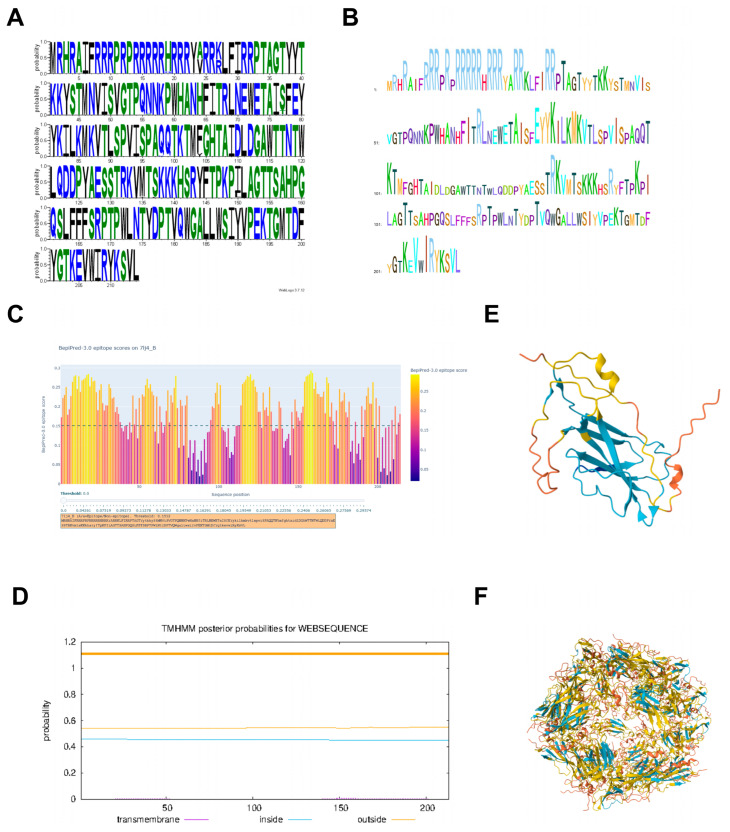
Bioinformatics analysis of PCV3 Cap. (**A**) Conservation analysis of amino acid sequence of PCV3 Cap. (**B**) Correlation analysis of amino acid nuclear localization of PCV3 Cap (**C**) Prediction of B-cell epitopes of PCV3 Cap. (**D**) Analysis of the transmembrane domain of PCV3 Cap. (**E**) Structure prediction of truncated PCV3 Cap monomer. (**F**) The predicted structure of truncated PCV3 Cap polymers.

Conservation analysis of 2403 PCV3 Cap sequences was conducted using MEGA 11.0 and WebLogo 3 (Figure 1A), in which the overall height of each residue stack represents the degree of sequence conservation at the corresponding position. The results revealed that A24V and R27K were the predominant mutation sites within the Cap, whereas the remaining amino acid residues were highly conserved. Subsequently, the NLS Explorer platform was employed to predict the nuclear localization potential of each residue within the conserved Cap sequence (Figure 1B), confirming that the arginine (R) residues at positions 1–32 constitute the core NLS. Prediction of B-cell epitopes using BepiPred 3.0 indicated that the amino acid region spanning positions 81–94 showed no overlap with the major predicted B-cell epitopes (Figure 1C). Furthermore, TMHMM analysis confirmed the absence of transmembrane domains within the Cap (Figure 1D). Based on these bioinformatic findings, the N-terminal 32 amino acids of the Cap were truncated to eliminate the arginine-rich NLS region that impedes soluble expression in *E. coli*, while preserving the majority of immunodominant epitopes. The monomeric and oligomeric structures of the truncated Cap were subsequently predicted using AlphaFold 3 (Figure 1E,F). Structural prediction suggests that the truncated Cap may have the propensity to self-assemble into virus-like particles (VLPs).

### 3.2. Cloning Results of Recombinant Plasmid pET-28a-PCV3-Cap

**Figure 2:**
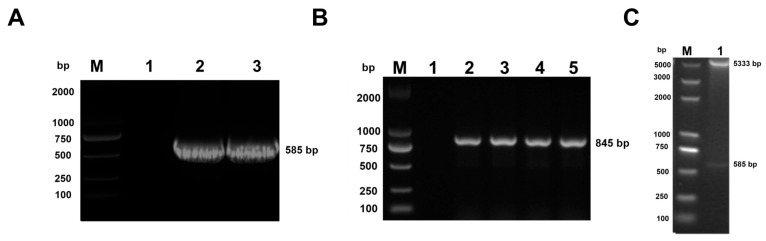
Construction of pET-28a-PCV3-Cap recombinant plasmid. (**A**) PCR amplification of PCV3 Cap gene. M: DL2000 DNA marker; Lane 1: negative control; Lanes 2–3: PCV3 Cap target gene. (**B**) PCR identification of pET-28a-PCV3-Cap bacterial solution. M: DL2000 DNA marker; Lane 1: negative control; Lanes 2–5: monoclonal clones of pET-28a-PCV3-Cap bacteria. (**C**) Enzyme digestion identification of pET-28a-PCV3-Cap recombinant plasmid, M: DL5000 DNA marker; Lane 1: pET-28a-PCV3-Cap recombinant plasmid. (See File S1).

The truncated PCV3 Cap gene was amplified by PCR (Figure 2A), yielding a target fragment of 585 bp, consistent with the expected size. The amplified fragment was subsequently ligated into the pET-28a vector to generate the recombinant plasmid pET-28a-PCV3-Cap. Following transformation into *E. coli* DH5αcompetent cells, the recombinant plasmid was identified by PCR using the universal primers T7 and T7ter. The resulting amplicon was 845 bp, in agreement with the predicted size (Figure 2B). The recombinant plasmid was further validated by double digestion with *EcoR I* and *Xho I* (Figure 2C). Finally, sequencing analysis confirmed the absence of mutations within the inserted gene fragment. Taken together, these results demonstrated the successful construction of the recombinant plasmid pET-28a-PCV3-Cap.

### 3.3. Soluble Expression and Purification of the Cap

**Figure 3:**
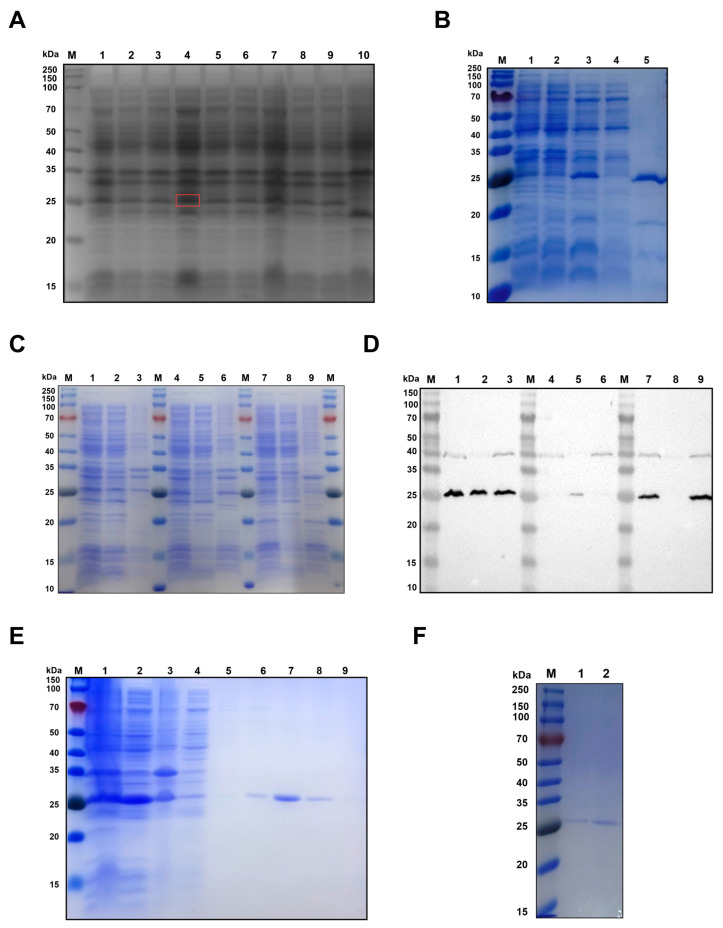
Expression of pET-28a-PCV3-Cap vector in *E. coli*. (**A**) Optimization of low-temperature induction conditions for BL21-pET-28a-PCV3-Cap. Lane M: protein molecular weight marker; Lanes 1–3: whole-cell lysates of BL21-pET-28a-PCV3-Cap induced at 16 °C for 16 h with 0.2, 0.4, and 0.8 mmol/L IPTG, respectively; Lanes 4–6: whole-cell lysates induced at 16 °C for 20 h with 0.2, 0.4, and 0.8 mmol/L IPTG, respectively; Lanes 7–9: whole-cell lysates induced at 16 °C for 24 h with 0.2, 0.4, and 0.8 mmol/L IPTG, respectively; Lane 10: uninduced BL21-pET-28a-PCV3-Cap control. The red box indicates the Cap expressed under the optimal induction conditions. (**B**) Solubility determination of the protein of BL21-pET-28a-PCV3-Cap, M: protein marker; Lane 1: BL21-pET-28a whole cells; Lane 2: uninduced BL21-pET-28a-Cap whole cells; Lanes 3–5: BL21-pET-28a-PCV3-Cap whole cells, supernatant, and pellet after lysis. (**C**) Identification of the solubility of SHuffle T7 Express *E. coli* B/Rosetta-gami2 (DE3) pLysS/Lemo21 (DE3)-pET-28a-PCV3-Cap by SDS-PAGE. M: protein marker; Lanes 1–3: SHuffle T7 Express *E. coli* B-pET-28a-PCV3-Cap whole cells, supernatant, and pellet after lysis; Lanes 4–6: Lemo21 (DE3)-pET-28a-PCV3-Cap whole cells, supernatant, and pellet after lysis; Lanes 7–9: Rosetta-gami2 (DE3) pLysS-pET-28a-PCV3-Cap whole cells, supernatant, and pellet after lysis. (**D**) Identification of the solubility of SHuffle T7 Express *E. coli* B/Rosetta-gami2 (DE3) pLysS/Lemo21 (DE3)-pET-28a-PCV3-Cap by Western blotting, M: protein marker; Lanes 1–3: SHuffle T7 Express *E. coli* B-pET-28a-PCV3-Cap whole cells, supernatant, and pellet after lysis, respectively; Lanes 4–6: Rosetta-gami2 (DE3) pLysS-pET-28a-PCV3-Cap whole cells, supernatant, and pellet after lysis, respectively; Lanes 7–9: Lemo21 (DE3)-pET-28a-PCV3-Cap whole cells, supernatant, and pellet after lysis, respectively. (**E**) Determination of the purification conditions for the recombinant Cap, M: protein marker; Lanes 1–3: supernatant and precipitate of the whole bacteria after lysis of SHuffle T7 Express *E. coli* B-pET-28a-PCV3-Cap; Lane 4: flow-through liquid; Lanes 5–9: elution solutions containing 50, 100, 200, 300, and 500 mmol/L imidazole, respectively. (**F**) Purification effect of recombinant Cap, M: protein marker; Lanes 1–2: purified Cap. (See File S1).

The recombinant plasmid pET-28a-PCV3-Cap was transformed into *E. coli* BL21 (DE3) for protein expression. Recombinant protein expression was induced with 0.2, 0.4, or 0.8 mmol/L IPTG at 16 °C for 16, 20, or 24 h, respectively. Coomassie Brilliant Blue staining revealed a distinct protein band of approximately 25 kDa in the induced samples compared with the empty-vector control. Based on band intensity, the optimal expression condition was determined to be induction with 0.2 mmol/L IPTG at 16 °C for 20 h (Figure 3A). Following induction, bacterial cells were harvested, disrupted by ultrasonication, and centrifuged to separate the supernatant and pellet fractions. Solubility analysis demonstrated that the Cap was predominantly present in the insoluble fraction (Figure 3B), indicating that the recombinant protein was mainly expressed as inclusion bodies.

To enhance the solubility of the recombinant Cap, the plasmid was further transformed into three engineered expression strains, including *E. coli* SHuffle T7 Express, Lemo21 (DE3), and Rosetta-gami2 (DE3) pLysS, which are designed to facilitate soluble protein expression. All strains were induced under the optimized conditions described above. The supernatant and pellet fractions were subsequently collected for solubility analysis (Figure 3C) and Western blotting (Figure 3D) analysis. The results showed that the Cap was expressed in a soluble form exclusively in *E. coli* SHuffle T7 Express. The soluble recombinant Cap present in the supernatant was further purified by nickel-affinity chromatography and eluted with 0.2 M imidazole (Figure 3E). Coomassie Brilliant Blue staining revealed a single prominent band at approximately 25 kDa in the purified fraction (Figure 3F), confirming the successful acquisition of highly purified recombinant Cap.

### 3.4. Serological Reactivity of Cap

**Figure 4:**
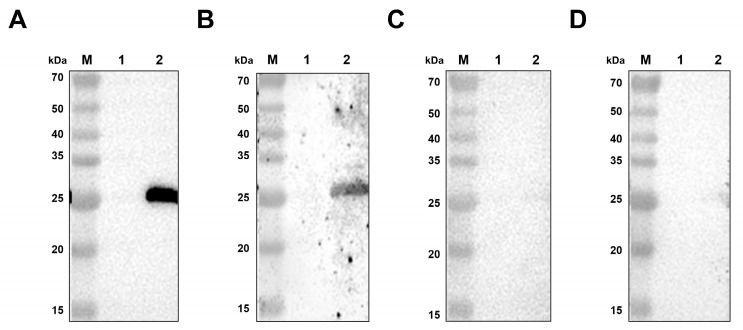
Recombinant Cap reactogenicity identification. (**A**) Recombinant Cap reacting with His-tag monoclonal antibody. (**B**) Recombinant Cap reacting with PCV3 clinically positive serum. (**C**) Recombinant Cap reacting with SPF pig negative serum. (**D**) Recombinant Cap reacting with PCV2 clinically positive serum, M: protein marker; Lane 1: SHuffle T7 Express *E. coli* B pET-28a lysate. Lane 2: recombinant Cap. (See File S1).

The antigenicity of the purified recombinant Cap was subsequently evaluated by Western blotting analysis using an anti-His monoclonal antibody, PCV3-positive serum, SPF pig serum, and PCV2-positive serum. The results demonstrated that the recombinant Cap was specifically recognized by both the anti-His monoclonal antibody (Figure 4A) and PCV3-positive serum (Figure 4B). In contrast, no detectable reactivity was observed with SPF pig serum (Figure 4C) or PCV2-positive serum (Figure 4D). Collectively, these findings verified the antigenic integrity and specificity of the purified recombinant Cap, confirming its ability to be specifically recognized by PCV3-positive clinical sera.

### 3.5. Optimization of PCV3 Cap Indirect ELISA Reaction Conditions

**Figure 5:**
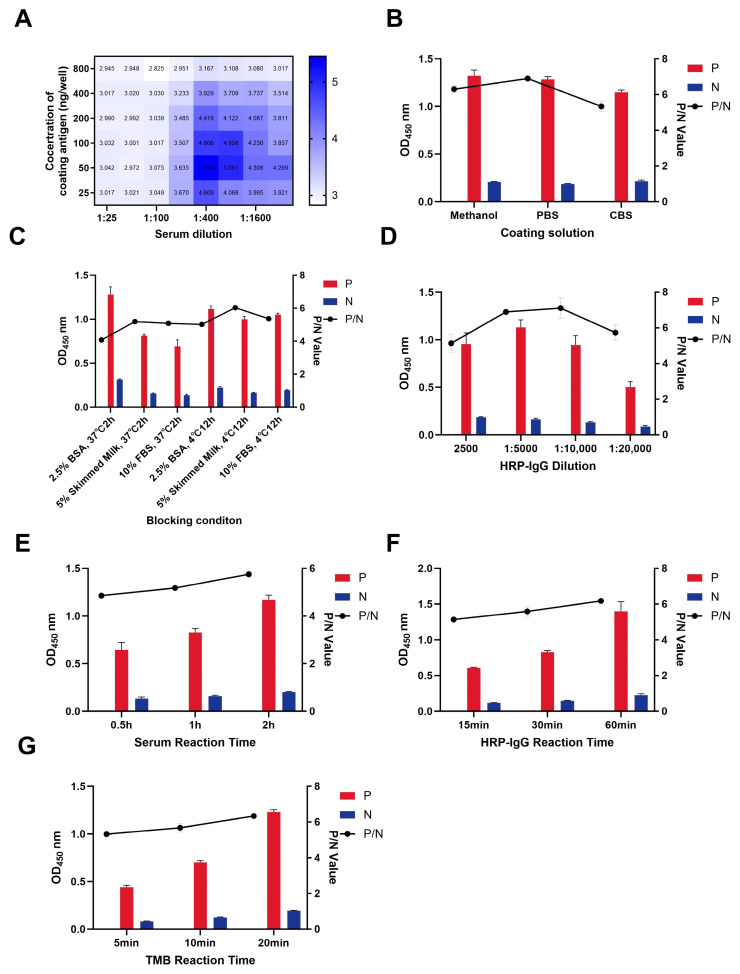
Optimization of ELISA reaction conditions. (**A**) Checkerboard titration for optimal coating antigen concentration and serum dilution. The heatmap shows P/N ratios, with darker shades representing higher ratios. (**B**–**G**) Screening of coating buffer (**B**), blocking condition (**C**), goat anti-pig IgG/HRP dilution (**D**), serum incubation time (**E**), goat anti-pig IgG/HRP incubation time (**F**), and TMB reaction time (**G**). Representative data from three independent experiments (mean ± SD) were analyzed with a two-tailed Student’s *t*-test. (See File S2).

The operational parameters of the indirect ELISA were optimized using a checkerboard titration strategy. The recombinant Cap was diluted to 0.5 mg/L in PBS, which was selected as the coating buffer, and used as the coating antigen (Figure 5A,B). Plates were blocked with 5% skimmed milk overnight at 4 °C (Figure 5C). Serum samples were diluted to 1:400 and incubated at 37 °C for 2 h (Figure 5A,E). Goat anti-pig IgG-HRP was diluted to 1:10,000 and incubated at 37 °C for 1 h (Figure 5D,F). In addition, the optimal incubation time for the TMB single-component substrate solution was determined to be 20 min (Figure 5G).

### 3.6. Determination of the Cut-Off Value for the Indirect ELISA

**Figure 6:**
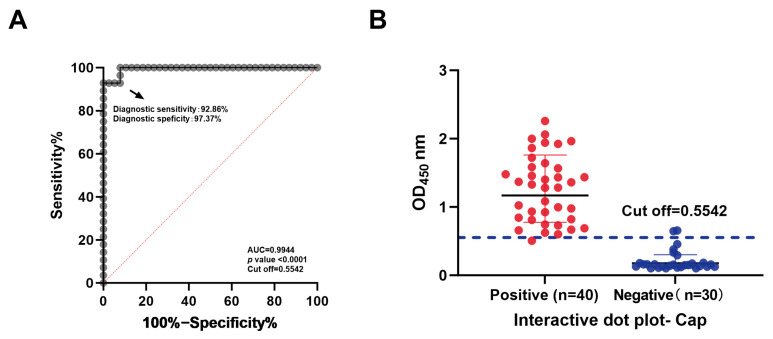
Determination of the cut-off values for the indirect ELISA using ROC curve analysis. (**A**) ROC curve analysis of the indirect ELISA for pig serum detection. (**B**) Distribution of OD_450_ for 30 negative and 40 positive pig serum samples detected by the indirect ELISA.

Following systematic optimization of the ELISA conditions, a panel of 40 PCV3-positive and 30 PCV3-negative swine serum samples was employed to determine the diagnostic cutoff value through receiver operating characteristic (ROC) curve analysis. The results indicated that the optimal cutoff value was 0.5542, corresponding to a sensitivity of 92.86% and a specificity of 97.37%, with a maximum Youden index of 90.23%. Furthermore, the assay exhibited excellent diagnostic performance, with an area under the ROC curve (AUC) of 0.9944 (95% confidence interval: 0.9838–1.000, *p* < 0.0001) (Figure 6A,B).

### 3.7. Evaluation of ELISA Assay Specificity, Sensitivity, and Reproducibility

**Figure 7:**
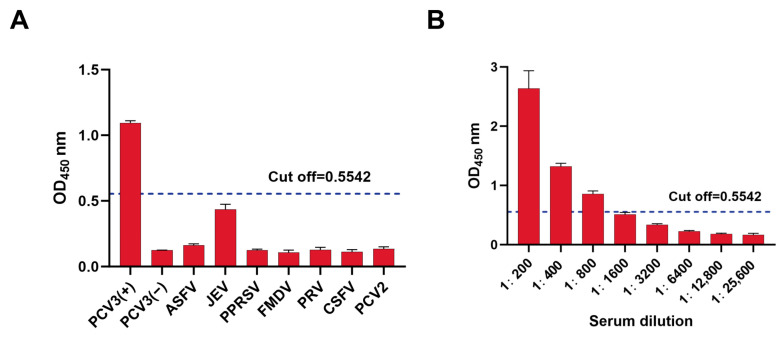
Evaluation of specificity and sensitivity of the indirect ELISA method. (**A**) Specificity testing of the ELISA assay using antibodies against other porcine disease pathogens, including CSFV, JEV, PRV, PCV2, ASFV, FMDV, and PRRSV. (**B**) Sensitivity testing of the ELISA assay using PCV3-positive sera. Representative data from three independent experiments (mean ± SD) were analyzed with a two-tailed Student’s *t*-test. (See File S2).

The diagnostic specificity of the indirect ELISA was assessed using sera positive for CSFV, JEV, PRV, PCV2, ASFV, FMDV, and PRRSV. All heterologous sera produced OD_450_ values below the established cutoff, indicating the absence of cross-reactivity with antibodies against these common swine viruses and confirming the high specificity of the assay (Figure 7A). The sensitivity of the assay was subsequently evaluated through twofold serial dilutions of PCV3-positive sera ranging from 1:200 to 1:25,600. The OD_450_ values remained above the cutoff threshold at a dilution of 1:800, demonstrating the adequate sensitivity of established ELISA (Figure 7B). To further assess assay consistency and stability, intra-assay and inter-assay variability were examined using three PCV3-positive and three PCV3-negative serum samples representing different antibody levels. The coefficients of variation (CVs) of the OD_450_ values ranged from 1.44% to 5.28% for intra-assay analyses and from 2.87% to 9.80% for inter-assay analyses, with all values remaining below 10% (Table 1). Collectively, these findings indicate that the established indirect ELISA demonstrates robust specificity, sensitivity, and reproducibility.

### 3.8. Clinical Application

A total of 144 clinical porcine serum samples collected from multiple pig farms were concurrently analyzed using the established indirect ELISA and the standard PCV3 PCR method (DB37/T 4047-2020) to evaluate the clinical applicability of the assay. The indirect ELISA identified 104 PCV3-positive samples, corresponding to a positivity rate of 72.22%, with 40 samples testing negative. In comparison, the PCR method detected 107 positive samples, yielding a positivity rate of 74.30%, and 37 negative samples. The positive concordance rate between the two methods was 84.11%, the negative concordance rate was 62.16%, and the overall concordance rate reached 78.47% (Table 2). These results indicate acceptable clinical agreement, suggesting that the established ELISA represents a practical and effective serological tool for PCV3 epidemiological surveillance.

## 4. Discussion

PCV3 is now globally disseminated, imposing a substantial economic burden on the swine industry [26]. The absence of a stable and efficient cell culture system for PCV3 propagation has substantially impeded investigations into viral pathobiology and vaccine development [7]. Under these circumstances, rigorous biosecurity management, coupled with timely diagnosis and sustained surveillance, remains the cornerstone of PCV3 prevention and control. Therefore, the establishment of reliable and cost-effective serological assays is of particular significance for monitoring viral circulation at the population level. In the present study, an indirect ELISA based on a soluble truncated PCV3 Cap was developed, providing a practical approach for large-scale serological surveillance of PCV3 infection.

To date, several ELISA platforms have been developed using PCV3 Cap produced in either prokaryotic or eukaryotic expression systems. Cao et al. reported that an NLS-truncated Cap, despite being expressed as inclusion bodies, retained the ability to self-assemble into VLPs and was subsequently employed as the coating antigen for an indirect ELISA detecting PCV3-specific antibodies [11]. Likewise, Zhang et al. utilized the baculovirus expression vector system to generate full-length Cap and established an indirect ELISA to investigate the PCV3 infection rate in serum samples from healthy sows and those with reproductive failure [15]. Nevertheless, the purification of inclusion body-derived proteins requires tedious denaturation and refolding procedures [27,28], whereas eukaryotic expression platforms are often associated with high manufacturing costs, low production efficiency, and extended processing periods [29,30]. Such drawbacks inevitably limit assay scalability and hinder their widespread application in routine epidemiological surveillance. One of the principal barriers to soluble expression of the PCV3 Cap in *E. coli* is its N-terminal NLS, which is enriched in arginine residues and rare codons and consequently interferes with proper protein folding [12]. Although truncation of the NLS region has proven effective in improving the soluble expression of Cap from other porcine circoviruses [31,32], its effectiveness for PCV3 remains controversial, with divergent outcomes reported in previous studies [33].

Based on the conservation profile of the PCV3 Cap, nuclear localization prediction, B-cell epitope mapping, and transmembrane domain analysis were further performed, leading to the selection of an N-terminally truncated Cap lacking the first 32 amino acids [12,34]. Notably, sequence conservation analysis revealed that the PCV3 Cap contains only two prevalent polymorphic sites, A24V and R27K [35,36], suggesting that the truncated antigen retains broad reactivity toward PCV3-specific antibodies and is therefore well suited for serological surveillance across different viral lineages. Interestingly, these two amino acid substitutions also constitute the major molecular markers used for PCV3 genotype classification [37]. Following expression screening in four *E. coli* strains, namely BL21 (DE3), SHuffle T7 Express, Lemo21 (DE3), and Rosetta- gami2 (DE3) pLysS, efficient soluble expression of the truncated Cap was achieved exclusively in SHuffle T7 Express. This engineered strain is designed to promote cytoplasmic disulfide bond formation [38,39], which may facilitate proper folding of the PCV3 Cap and reduce protein aggregation. Although AlphaFold3 modeling predicted that the truncated Cap retained the capacity to self-assemble into VLPs, this prediction was not experimentally validated by transmission electron microscopy. Furthermore, the immunogenic properties of the truncated protein warrant additional investigation.

Given that PCV3 vaccines remain at the experimental stage and no commercial vaccine is currently available for field application [7], PCV3-specific antibodies detected in swine sera are presumed to arise exclusively from natural infection. Consistent with the findings of Zhang et al., who reported a strong correlation between PCV3 antigen and antibody detection in animals with high-level viremia [15], qPCR was employed to define the infection status of 70 serum samples used for assay validation. ROC curve analysis based on these well-characterized samples established an optimal cutoff value of 0.5542, with an AUC of 0.9944, a sensitivity of 92.86%, and a specificity of 97.37%, demonstrating excellent diagnostic performance comparable to previously reported assays. Subsequent evaluations of analytical specificity, sensitivity, and reproducibility further substantiated the robustness and reliability of the established ELISA.

The frequent occurrence of mixed infections, subclinical infections, and persistent infections presents considerable challenges for the accurate diagnosis of PCV3 in field conditions. Consequently, effective serological surveillance remains essential for monitoring viral circulation and guiding disease control strategies. In the analysis of 144 clinical serum samples, the newly developed ELISA yielded a positive detection rate of 72.22%, whereas the reference PCR assay detected 74.30% positivity. As the two methods target distinct biological indicators—host antibody responses and viral nucleic acids, respectively—the comparable detection rates observed in this study further reflect the widespread prevalence of PCV3 infection in commercial pig populations. Notably, the overall agreement between the two assays reached 78.47%, with positive and negative agreement rates of 84.11% and 62.16%, respectively. The observed discrepancies may be attributable to antibody levels below the detection threshold in animals carrying low viral loads or at specific stages of infection [40]. Therefore, further investigations are warranted to assess the diagnostic performance of the established ELISA in serum samples with low-level viremia.

## 5. Conclusions

In conclusion, soluble expression of the PCV3 Cap was successfully achieved through N-terminal (aa 1–32) truncation and expression in the SHuffle T7 *E. coli* system, effectively overcoming the long-standing limitation of inclusion body formation in prokaryotic expression. Based on this purified soluble antigen, a novel indirect ELISA was developed, exhibiting high specificity, sensitivity, and reproducibility. The assay demonstrated satisfactory concordance with the standard PCR method in clinical evaluation and is well-suited for rapid, high-throughput serological surveillance of PCV3. Collectively, this study provides a reliable and practical diagnostic tool for PCV3 epidemiological monitoring and disease control.

## Figures and Tables

**Table 1:** Reproducibility of the indirect ELISA assay validated using pig serum.

Samples	Intra-Coefficient of Variation (n = 3)			Inter-Coefficient of Variation (n = 3)		
	$\bar{X}$	SD	CV (%)	$\bar{X}$	SD	CV (%)
Positive1	1.091	0.018	1.64	1.113	0.041	3.65
Positive2	1.552	0.040	2.58	1.594	0.082	5.14
Positive3	2.069	0.042	2.04	2.131	0.104	4.88
Negative1	0.155	0.008	5.28	0.162	0.016	9.80
Negative2	0.113	0.002	1.44	0.116	0.005	3.96
Negative3	0.124	0.002	1.64	0.126	0.004	2.87

**Table 2:** Detection of clinical pig serum samples and comparison with the Chinese regional standard PCR method.

Indirect ELISA Method	Standard PCR Method			
	Positive	Negative	Total	Agreement Rate (%)
Positive	90	14	104	84.11
Negative	17	23	40	62.16
Total	107	37	144	78.47

## Data Availability

The original contributions presented in this study are included in the article/Supplementary Material. Further inquiries can be directed to the corresponding author.

## Supplementary Materials

The following supporting information can be downloaded at: https://www.mdpi.com/article/10.3390/vetsci13070704/s1, File S1: Original Western blot and PCR images. File S2: Original data corresponding to Figure 5A–G and Figure 7A,B.
