# Supplementary figures and images for: Establishment of an Indirect ELISA Detection Method for Porcine Circovirus 3 Based on Soluble Cap Protein
Source: Vet Sci. 2026 Jul 18;13(7):704. doi: 10.3390/vetsci13070704 (PMC13431520; doi:10.3390/vetsci13070704)

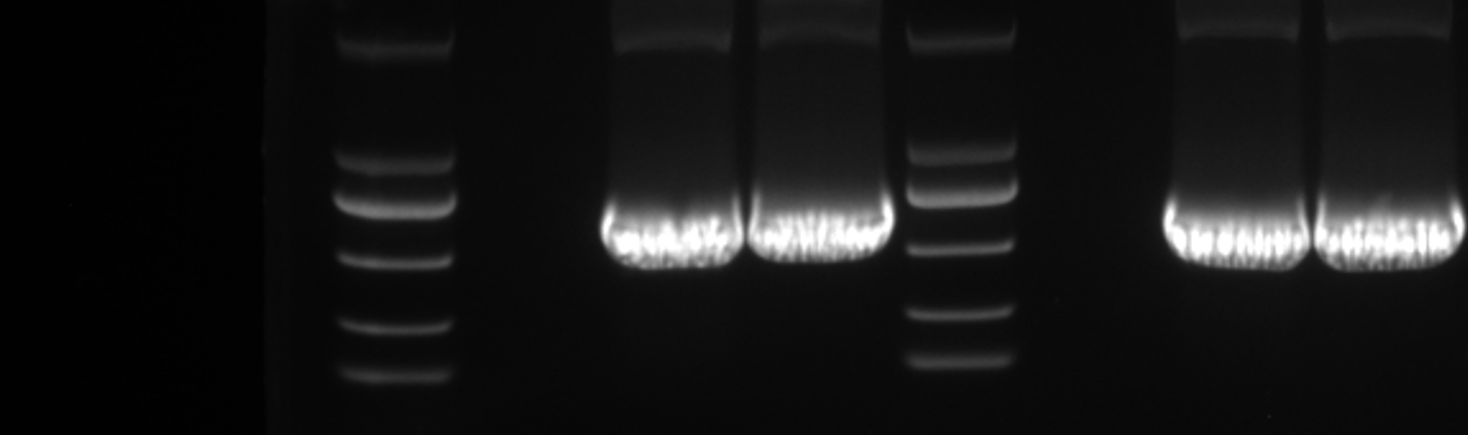

Supplement: Supplementary file 1 [file vetsci-13-00704-s001.zip › File S1/1.tif]

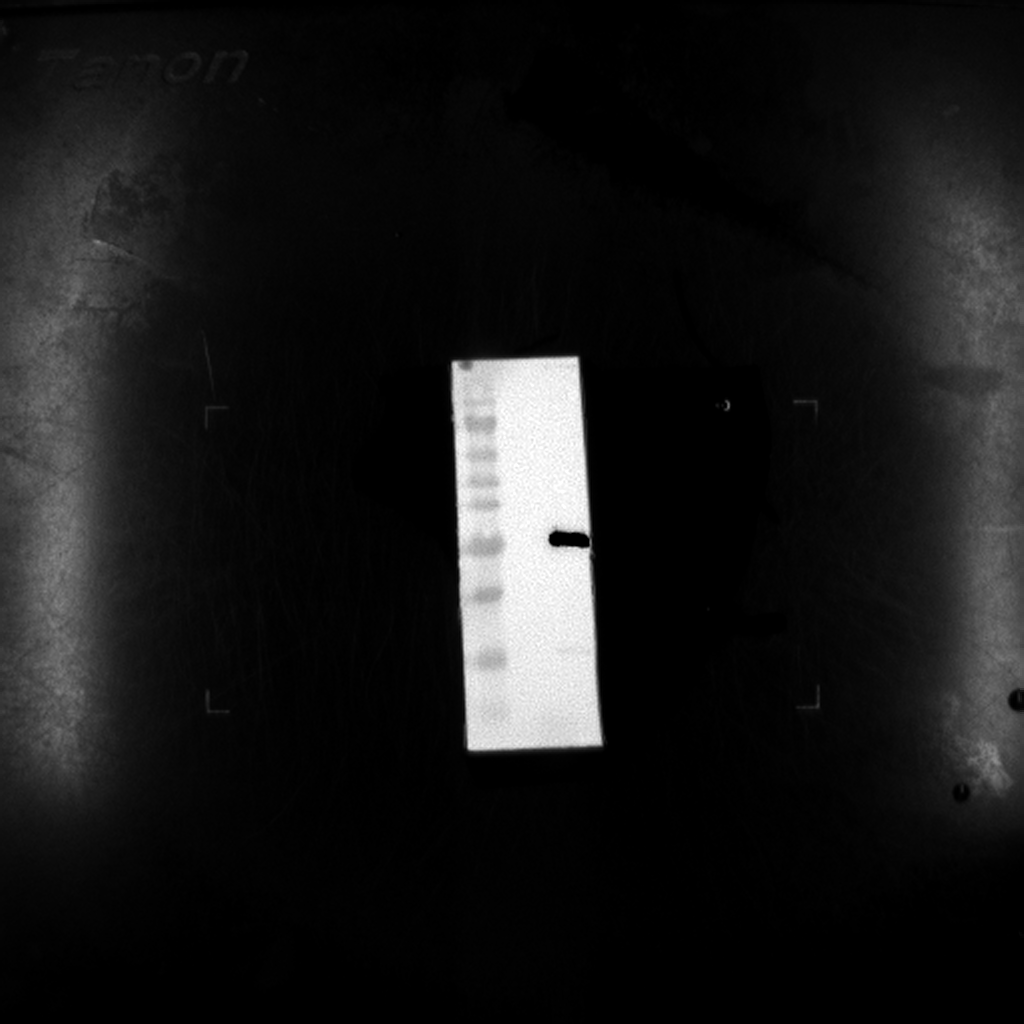

Supplement: Supplementary file 1 [file vetsci-13-00704-s001.zip › File S1/10.tif]

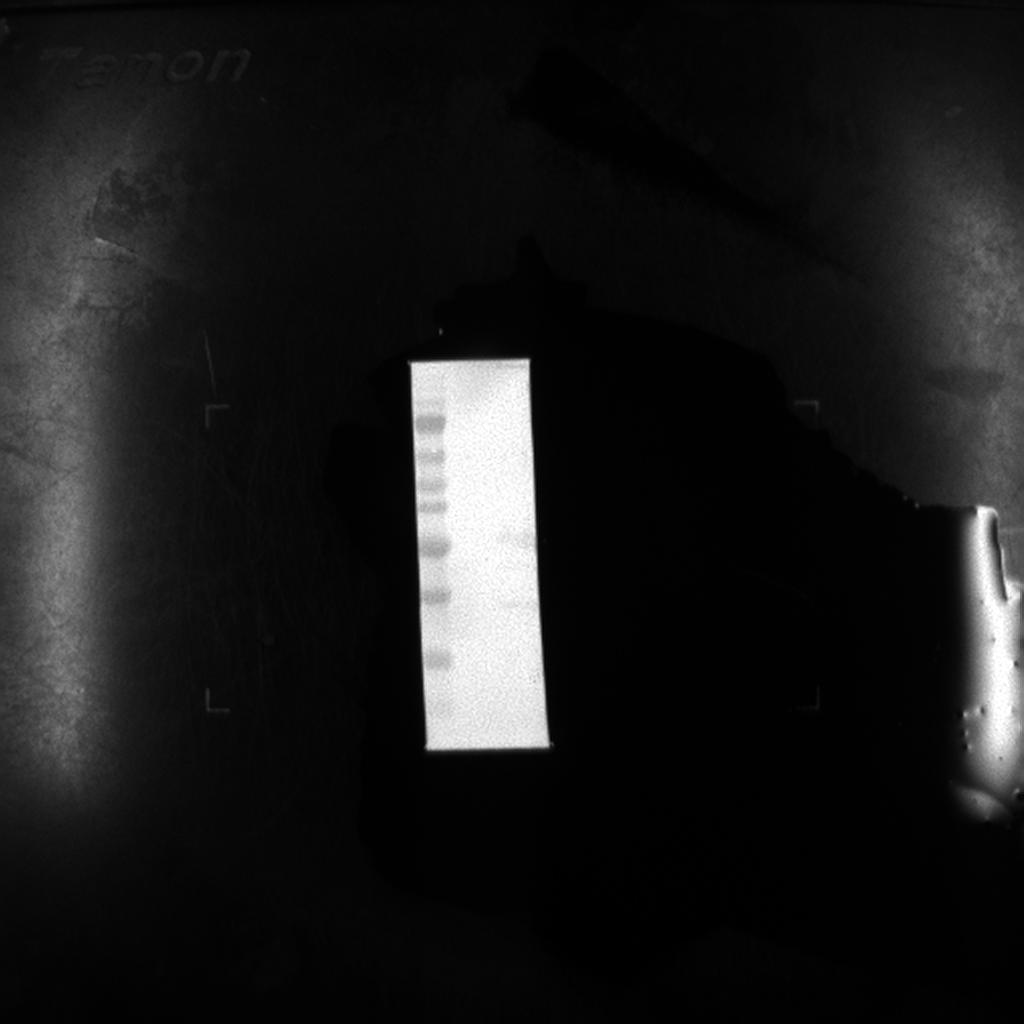

Supplement: Supplementary file 1 [file vetsci-13-00704-s001.zip › File S1/11.tif]

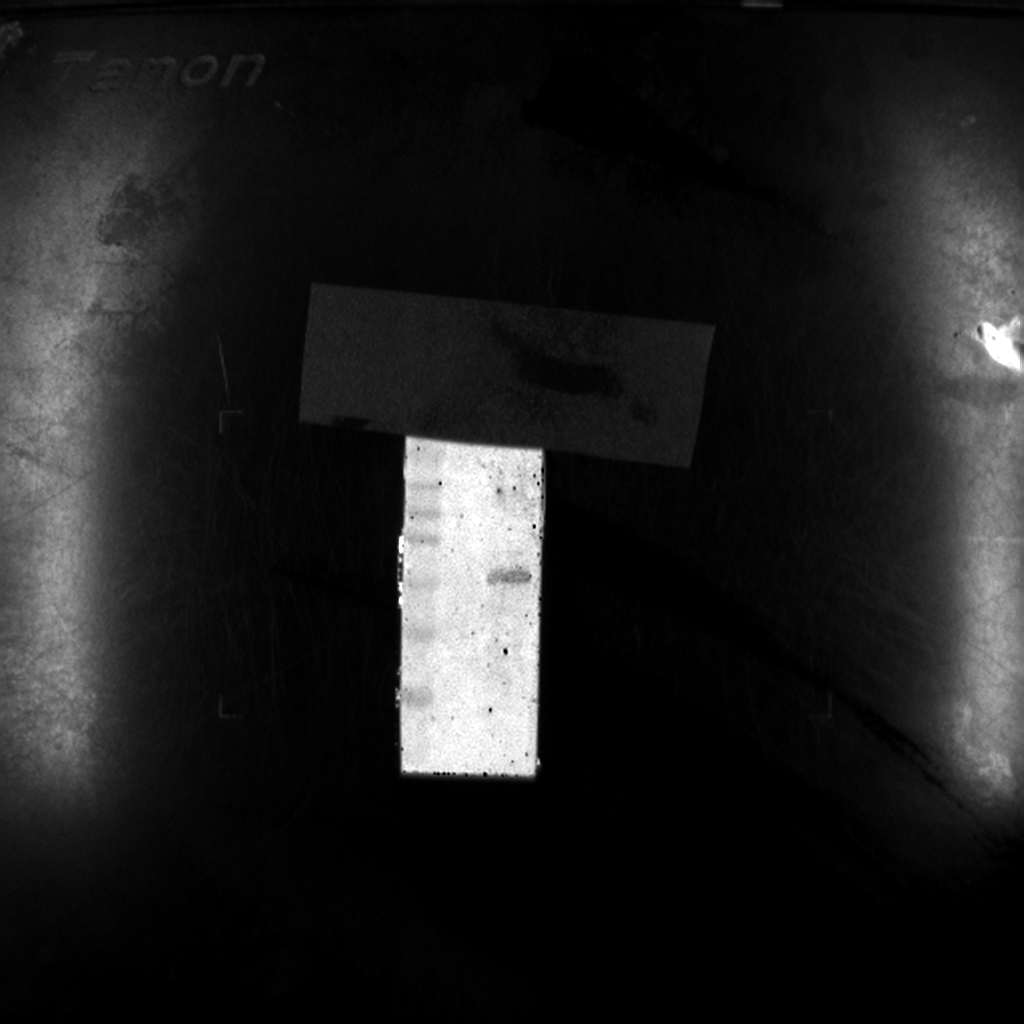

Supplement: Supplementary file 1 [file vetsci-13-00704-s001.zip › File S1/12.tif]

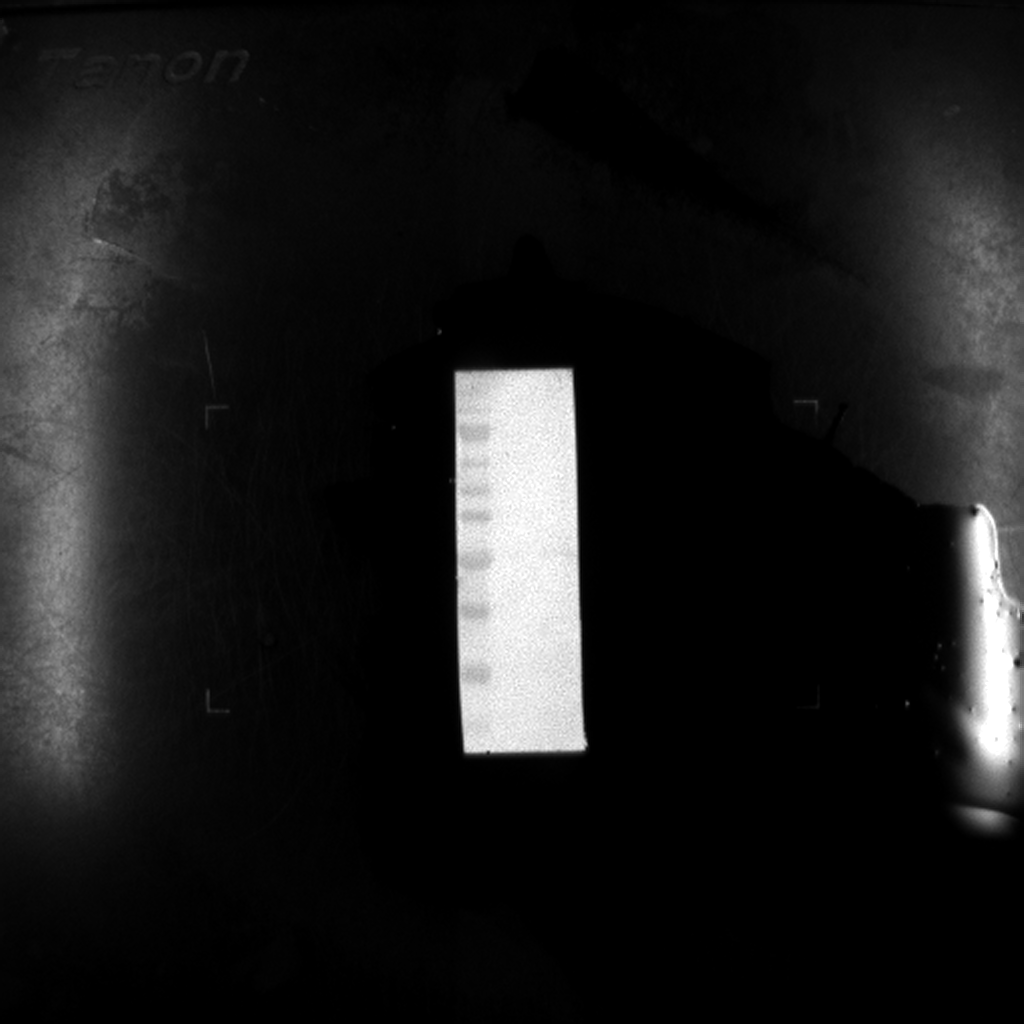

Supplement: Supplementary file 1 [file vetsci-13-00704-s001.zip › File S1/13.tif]

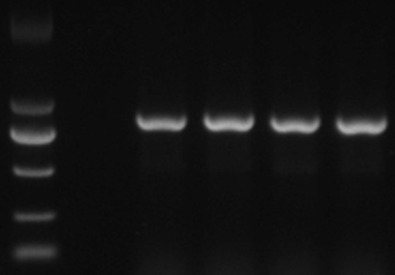

Supplement: Supplementary file 1 [file vetsci-13-00704-s001.zip › File S1/2.tif]

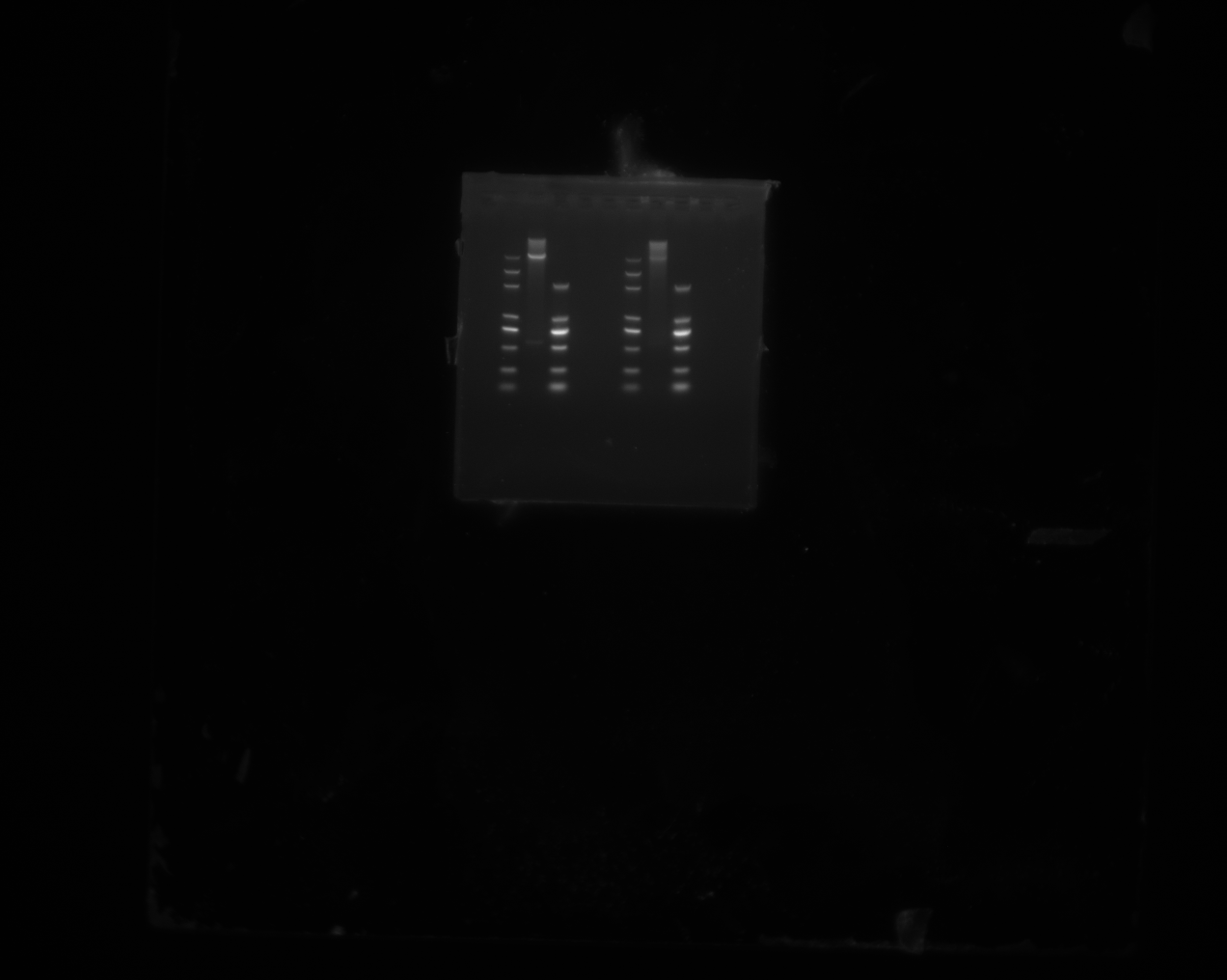

Supplement: Supplementary file 1 [file vetsci-13-00704-s001.zip › File S1/3.tif]

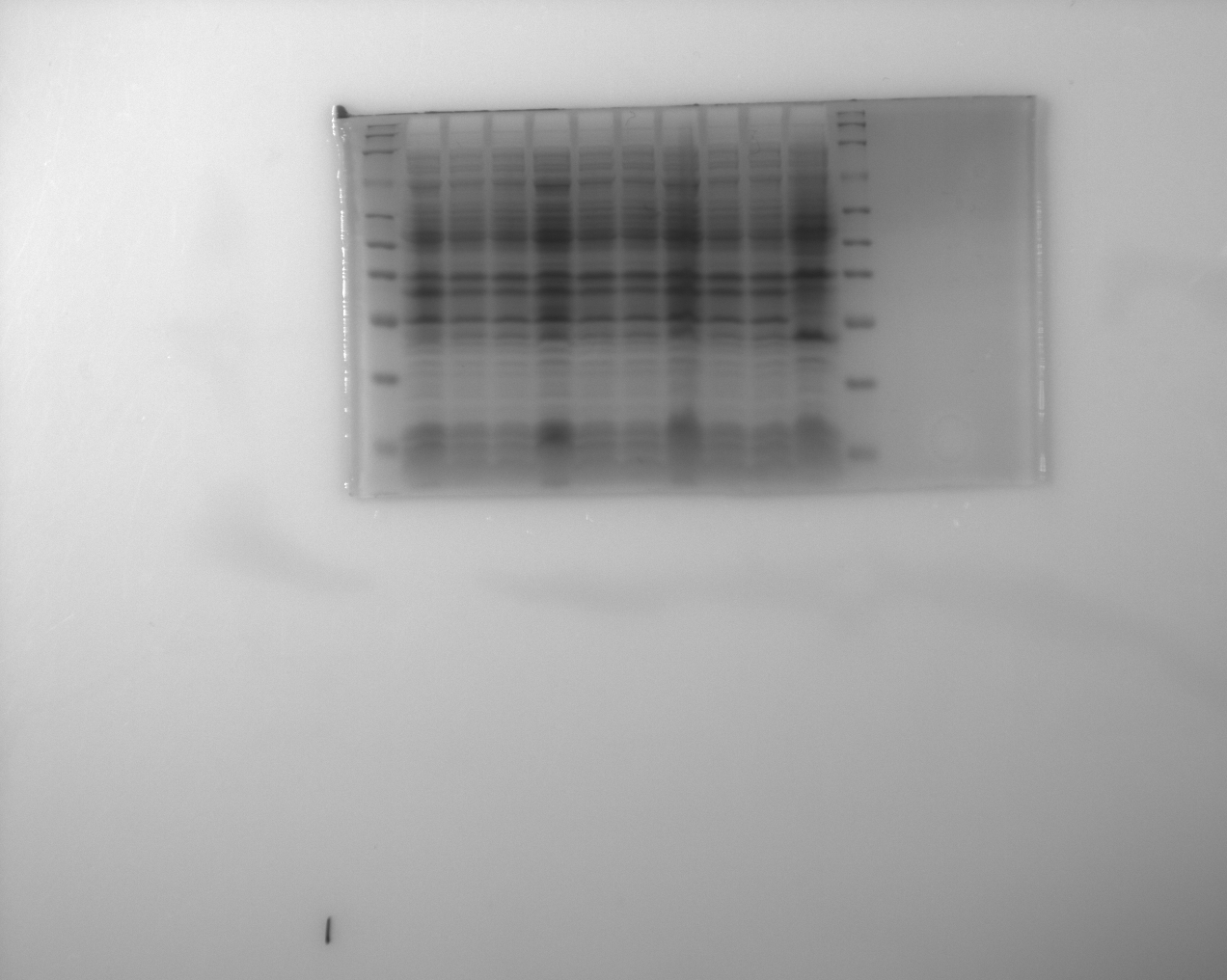

Supplement: Supplementary file 1 [file vetsci-13-00704-s001.zip › File S1/4.tif]

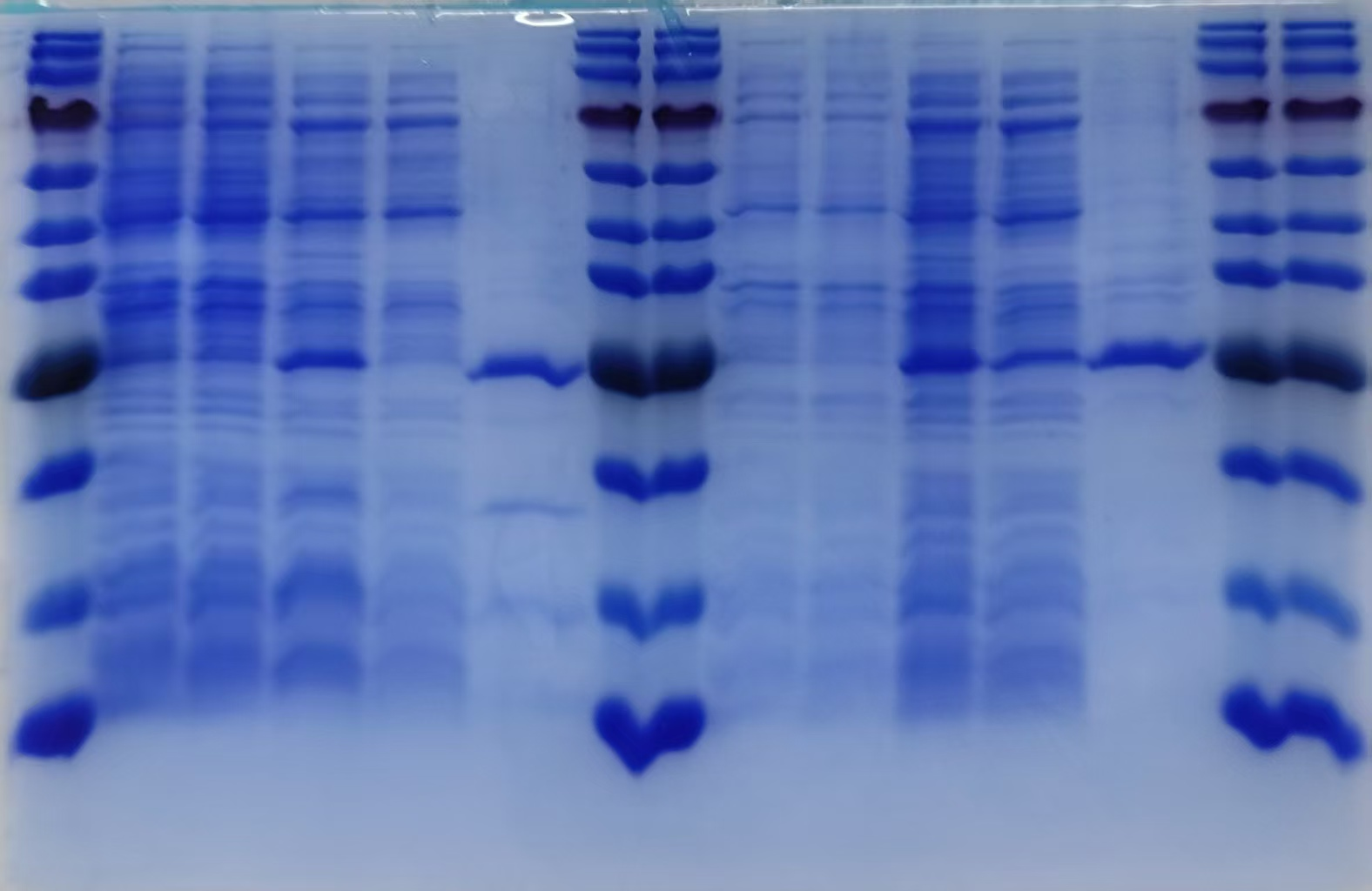

Supplement: Supplementary file 1 [file vetsci-13-00704-s001.zip › File S1/5.tif]

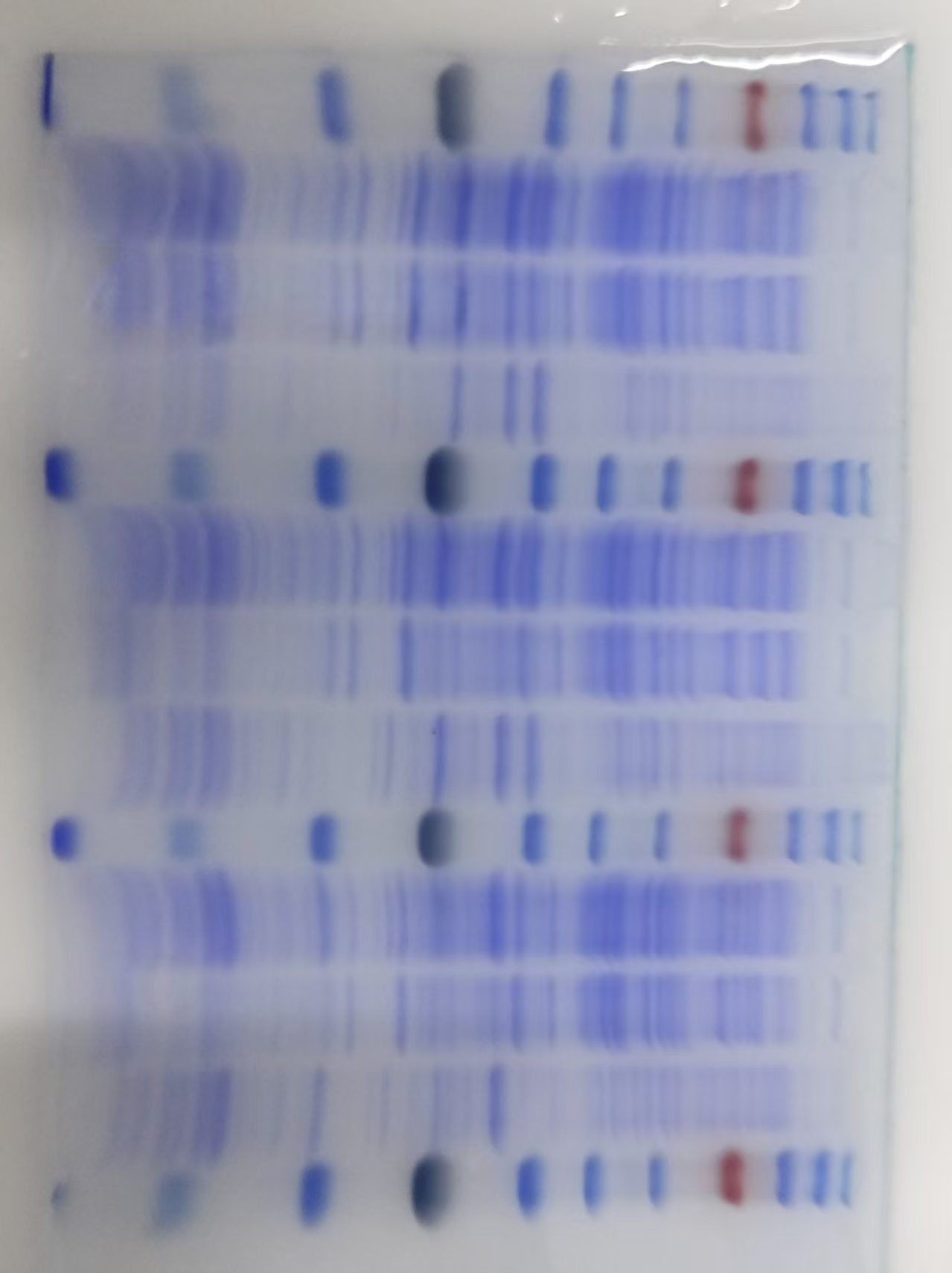

Supplement: Supplementary file 1 [file vetsci-13-00704-s001.zip › File S1/6.tif]

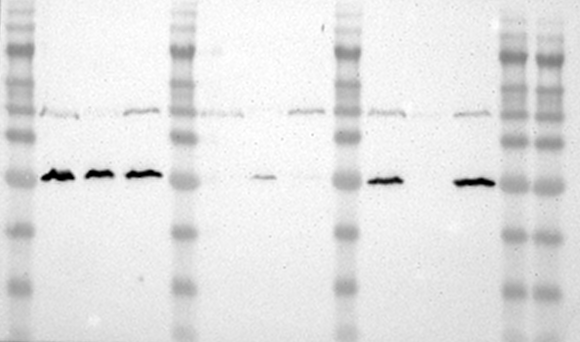

Supplement: Supplementary file 1 [file vetsci-13-00704-s001.zip › File S1/7.tif]

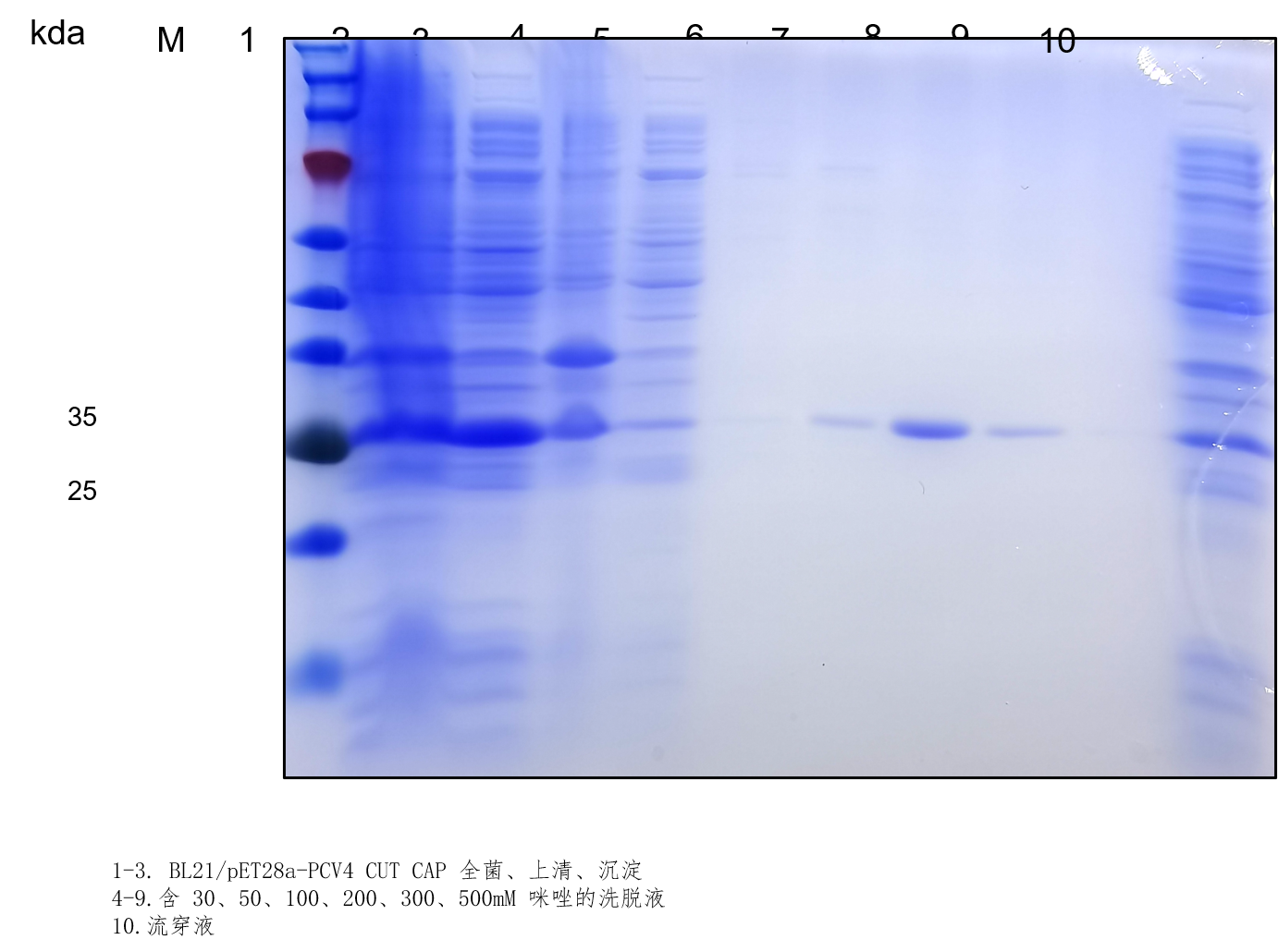

Supplement: Supplementary file 1 [file vetsci-13-00704-s001.zip › File S1/8.tif]

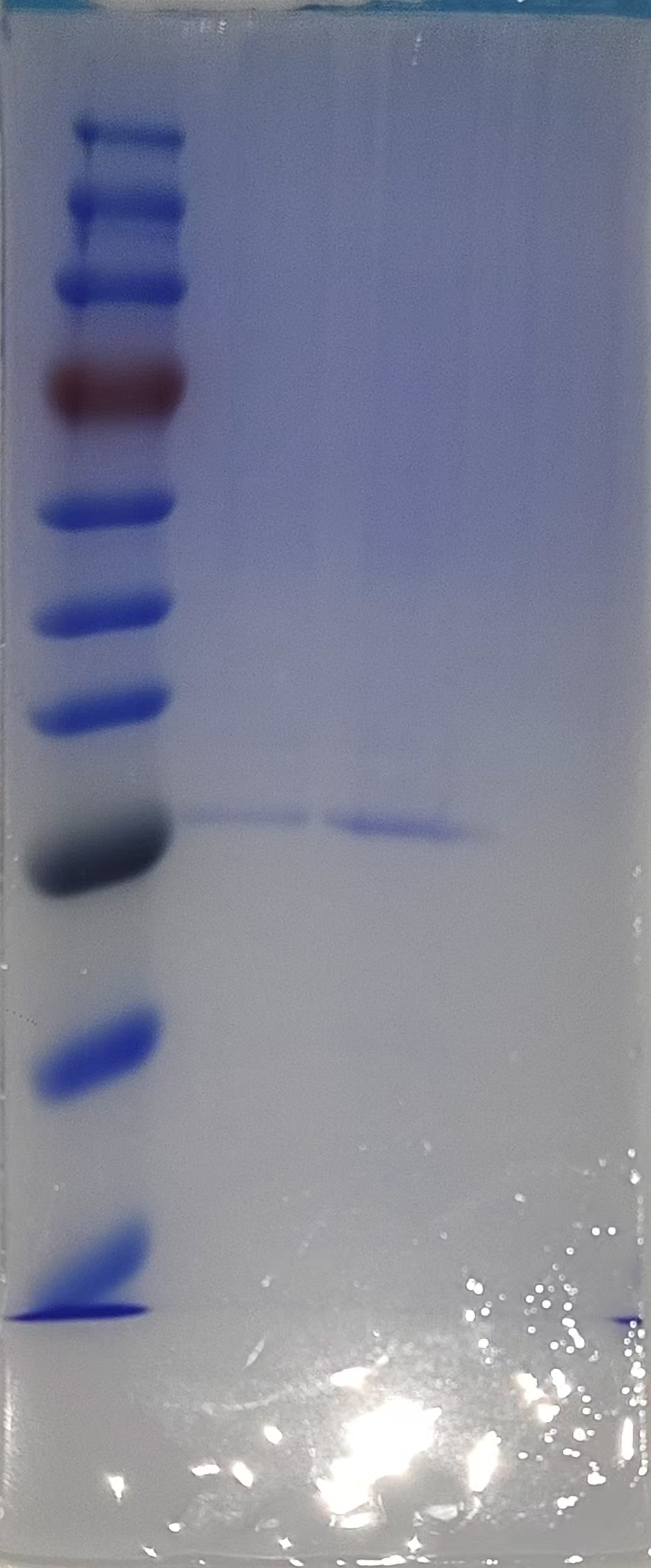

Supplement: Supplementary file 1 [file vetsci-13-00704-s001.zip › File S1/9.tif]
